# The Relation Between Gender Egalitarian Values and Gender Differences in Academic Achievement

**DOI:** 10.3389/fpsyg.2020.00236

**Published:** 2020-02-20

**Authors:** Kimmo Eriksson, Marie Björnstjerna, Irina Vartanova

**Affiliations:** ^1^School of Education, Culture and Communication, Mälardalen University, Västerås, Sweden; ^2^Centre for Cultural Evolution, Stockholm University, Stockholm, Sweden; ^3^Institute for Futures Studies, Stockholm, Sweden

**Keywords:** gender egalitarian values, gender equality, gender differences, academic achievement, mathematics education, literacy abilities

## Abstract

Gender differences in achievement exhibit variation between domains and between countries. Much prior research has examined whether this variation could be due to variation in gender equality in opportunities, with mixed results. Here we focus instead on the role of a society’s values about gender equality, which may have a more pervasive influence. We pooled all available country measures on adolescent boys’ and girls’ academic achievement between 2000 and 2015 from the Program for International Student Assessment (PISA) and Trends in International Mathematics and Science Study (TIMSS) assessments of math, science, and reading. We then analyzed the relation between gender differences and country levels of gender egalitarian values, controlling for country levels of living standards and indicators of gender equality in opportunities. Gender egalitarian values came out as the most important predictor. Specifically, more gender egalitarian values were associated with improved performance of boys relative to girls in the same countries. This pattern held in reading, where boys globally perform substantially worse than girls, as well as in math and science where gender differences in performance are small and may favor either boys or girls. Our findings suggest a previously underappreciated role of cultural values in moderating gender gaps in academic achievement.

## Introduction

The rise in gender egalitarian values in industrialized and postindustrial societies has had wide-ranging effects (Inglehart and Norris, 2003). It is now a nearly universal phenomenon that girls outperform boys in school (Voyer and Voyer, 2014) and that gender differences in grade progression and school dropout favor girls—even in developing countries (Grant and Behrman, 2010). Many researchers are concerned about boys falling behind in school (Morris, 2012; DiPrete and Buchmann, 2013). However, gender differences in achievement vary with academic domains. This is especially well documented with respect to performance on standardized tests in the domains of reading and mathematics; the average girl always outperforms the average boy at reading, but in some countries, the opposite relation holds for math (Stoet and Geary, 2013). In other words, the gender difference in reading performance consistently favors girls, whereas the direction of the gender difference in math performance varies between countries (and may also vary over time within a country). In this paper, we will always calculate gender differences by taking the achievement of boys minus the achievement of girls, and we shall refer to this difference as *the relative achievement of boys vs. girls*. A negative value of the relative achievement of boys vs. girls thus signifies a gender difference that favors girls.

Could it be that the relative achievement of boys vs. girls in a country depends on the level of gender equality? This idea has been around for several decades (Baker and Jones, 1993). It has been examined in a number of studies using cross-national datasets on achievement on standardized tests, such as Program for International Student Assessment (PISA). A ground-breaking study by Guiso et al. (2008) found that the relative math achievement of boys vs. girls in 2003 PISA was negatively associated with country-level indicators of gender equality in opportunities. However, this finding has not been replicated in other waves of PISA (Stoet and Geary, 2013). Results vary across waves due to varying country samples as well as sampling error within countries. From the total evidence, it is unclear whether there is any robust association between the relative math achievement of boys vs. girls and gender equality in opportunities.

The purpose of this paper is to shift attention from gender equality in opportunities to gender egalitarian values. Whereas the former refers to outcome measures such as the relative participation of women and men in the workforce or in politics, the latter refers to cultural attitudes regarding the value of the genders: Are women and men equally important? Are they equally competent? Should they have the same rights? We will argue that such cultural values are likely to have a direct influence on the academic efforts of adolescent girls and boys, thereby shaping gender differences in achievement.

The potential role of gender egalitarian values has largely been neglected in prior research on gender differences in achievement. In the above-mentioned analysis of 2003 PISA data, a measure of gender egalitarian values was included alongside a measure of gender equality in opportunities (Guiso et al., 2008). Both measures were found to be negatively associated with the relative math achievement of boys vs. girls, but the effect was not statistically significant for the measure of gender egalitarian values. Subsequent research has focused exclusively on the effect of gender equality in opportunities.

There are two reasons why we decided to revisit the role of gender egalitarian values. First, there are theoretical reasons to believe that gender egalitarian values may affect the schoolwork of girls and boys. Second, there are empirical reasons to doubt the robustness of the original findings. Subsequent research has shown that patterns of gender differences in achievement in the 2003 PISA dataset do not tend to replicate in other waves of PISA (Stoet and Geary, 2013).

### Our Methodological Approach

An important question is why different waves of PISA and Trends in International Mathematics and Science Study (TIMSS) would yield differing patterns of gender differences in achievement. The time between consecutive waves of PISA and TIMSS is just 3 and 4 years, respectively. It seems unlikely that gender differences change much in such a short time. However, the *measures* of gender differences may still change due to sampling error. The size of student samples in these assessments is usually around 5000 per country, see the official reports of the methods used in TIMSS and PISA (Mullis et al., 2009; Schleicher et al., 2009). These are large samples and consequently the standard errors of mean scores are small—but not negligible. Descriptive statistics of data from TIMSS and PISA are easily available from the data explorer service provided by the National Center for Education Statistics^1^. On the scoring scale that is used (which has a global mean of 500), the standard error for country measures of gender differences is around four points. At the same time, the standard deviation of gender differences *across* countries is only around 10 points. Thus, although the measurement error at the country level is small, it is sufficiently large for an estimation of between-country variation in a single wave to be unreliable. Another issue is that the *sample of countries* choosing to participate in PISA or TIMSS varies between waves. These samples are of limited size (e.g., 40 countries in 2003 PISA) and not representative of all countries that potentially could participate. A correlation may come out quite differently when estimated in different non-representative samples. Both these sampling issues can be alleviated by pooling the data from many years. We then obtain a larger sample of countries as well as several measures of gender differences in each country. These can then be analyzed by multi-level methods, nesting country-years in countries.

### Background

#### Gender Differences in Academic Achievement

There is an extensive empirical literature on gender differences in academic achievement. While girls have historically often been disadvantaged, they are now surpassing boys in rate of school enrollment and grade completion even in many developing countries (Grant and Behrman, 2010). In modern times, the big picture is that girls tend to do better than boys in school (Voyer and Voyer, 2014), with differences tending to be more pronounced in minority groups, in urban areas, and among students from families with low socioeconomic status (Morris, 2012). To explain these observations, several theories about the impact of individual and contextual factors have been put forward. Although men and women seem to be similar on most psychological variables (Hyde, 2005), it has been argued that girls on average have superior performance on some behavioral skills that are of importance for academic success, such as self-discipline (Duckworth and Seligman, 2006). Boys, on the other hand, more often express aggressive behaviors and display more developmental difficulties and negative attitudes toward learning (Zill and West, 2001; Lansford et al., 2012). It could therefore be argued that girls, in general, more easily adjust to the school environment. Gender norms have also been suggested to play a role; specifically, aspects of expressed masculinity might negatively affect boys’ achievement (Morris, 2012).

Gender differences in academic performance vary somewhat from kindergarten and up to high school (Robinson and Lubienski, 2011). In the present paper, we focus on gender differences among adolescents, where the best data is available. Importantly, gender differences also depend on whether performance is measured in terms of grades awarded in school or in terms of scores on standardized tests. In brief, boys are at a clear disadvantage when it comes to grades awarded, but perform relatively better at achievement tests (Duckworth and Seligman, 2006). If we look at achievement tests in mathematics, boys even perform somewhat better than girls in many countries; at reading tests, however, girls seem to outperform boys everywhere (Stoet and Geary, 2013). Our focus in this paper is on standardized tests in reading, math, and science.

#### How Gender Differences in Achievement Vary Between Countries

Most studies of the above-mentioned research on gender differences in school performance have taken place in western countries, especially in the United States. However, there is also an extensive literature on how gender differences vary between countries. For instance, a comprehensive cross-national meta-analysis on gender differences in school grades found that the size of the gender gap depended on whether studies covered North America, or Scandinavia, or the rest of the world (Voyer and Voyer, 2014). Studies where gender differences are measured using the same method in many countries are particularly informative. The foremost example is PISA, a large-scale assessment that has been conducted with a new wave every three years since 2000. PISA tests mathematics, science, and reading literacy among representative samples of 15-year-olds, mainly in OECD countries.

The first two major studies of gender differences in reading and mathematics based on PISA data came out the same year but used data from different waves, either the 2000 wave (Marks, 2008) or the 2003 wave (Guiso et al., 2008). Both studies found that the levels of relative achievement of boys vs. girls in mathematics and reading were very strongly correlated (i.e., in those countries where boys did particularly well in math relative to girls, they also did particularly well in reading). However, the studies reached somewhat different conclusions with respect to the role of gender equality. The first study examined the proportion of women in the workforce and found that it was “not associated with the gender gaps in mathematics” (Marks, 2008, p. 89). The second study examined the same measure of female participation in the workforce, as well as a measure of female political empowerment and a measure of gender egalitarian values, and concluded that “the gender gap in math scores disappears in countries with a more gender-equal culture” (Guiso et al., 2008, p. 1164). The latter conclusion was upheld also in a later analysis of the same 2003 PISA dataset (Else-Quest et al., 2010). However, it was not supported in subsequent analyses of the 2009 PISA data (Kane and Mertz, 2012; Reilly, 2012). Later detailed studies of all four waves of PISA from 2000 to 2009 confirmed that different waves yield results that point in different directions with respect to the role of gender equality (Stoet and Geary, 2013, 2015).

Another source of cross-national data on achievement in math and science is TIMSS. TIMSS tests eighth graders on what is covered by curricula, with a new wave every fourth year since 1995. Also research on TIMSS data has given a mixed picture of the relation between gender equality and gender differences in achievement. Kane and Mertz (2012) found a measure of gender equality to correlate either positively or negatively with the relative math achievement of boys vs. girls, depending on whether the 2003 or 2007 TIMSS data were used. Other researchers using the 2011 TIMSS data found a null correlation (Reilly et al., 2019).

In sum, prior research suggests two important conclusions on how gender differences in math achievement vary between countries. First, gender differences in math achievement seem to be robustly linked to gender differences in reading achievement. Second, there does *not* seem to be a robust link between gender differences in academic achievement and country levels of gender equality.

### Theories and Research Questions

#### Theories on How Gender Equality Could Shape Gender Differences in Achievement

There are several theories on how the level of gender equality in a society could influence the gender difference in academic achievement. One idea is that gender segregation, specifically with respect to job opportunities, influence students’ motivation. This idea, known as the gender stratification hypothesis, has mainly been applied to achievement in mathematics: female students may do less well in math if there are less opportunities to jobs that require math skills for women than for men (Baker and Jones, 1993; Else-Quest et al., 2010). An alternative idea is that gender equality and prosperity are conditions that give individuals more freedom to pursue their intrinsic interests. To the extent that boys have a greater intrinsic interest in mathematics than girls do, boys would then perform relatively well at math in countries with greater gender equality and prosperity (Stoet et al., 2016). Note that these ideas lead to opposite predictions about the relation between gender equality and gender differences in math achievement, but none of them seem to account for the robust association between gender differences in reading and gender differences in mathematics achievement. A third idea is that gender differences in academic achievement (both in reading and math) may vary due to policy differences with respect to gender and education (Marks, 2008). However, gender differences in enrollment do not consistently predict gender differences in achievement (e.g., Else-Quest et al., 2010). In sum, none of the three ideas has received consistent support across the published analyses of PISA and TIMSS data.

#### The Case for Examining Gender Egalitarian Values

The above-mentioned theories all focus on gender equality in opportunities. A society’s underlying values with respect to gender equality may be an alternative driver of boys’ and girls’ relative academic achievement (Nollenberger et al., 2016). Here we elaborate on why, taking as our starting point Bronfenbrenner’s ecological perspective on human development (Bronfenbrenner, 1979, 2005). In brief, Bronfenbrenner’s model views children’s development as shaped through a dynamic interaction between the child and its environment. The environment has multiple layers, from the closest layer consisting of the child’s family, friends, and teachers to the outermost layer consisting of the society and culture in which the child lives. The surrounding culture may influence a child’s academic achievement through its influence on how the child interacts with family, friends, and teachers. We shall now outline various pathways by which gender egalitarian values could influence the relative achievement of boys vs. girls. We distinguish mechanisms that would yield a positive influence from those that would yield a negative influence. Our data will not allow us to test any of these mechanisms; the point of this section is demonstrating that there are plausible ways in which academic achievement could be influenced by cultural values rather than by expectations of future opportunities.

Attitudes toward gender could play a major role in what *parents* allow their children to do. This may be important for academic achievement, because students may put less effort into their studies if they have extra-curricular activities such as jobs, sports, and dating (Stevenson et al., 1993). Parental constraints on out-of-home activities for adolescents may be stricter for girls than for boys, in particular in cultural groups with less gender egalitarian values (Carrington et al., 1987). As values become more gender egalitarian we expect parental constraints on girls to become more relaxed. As the range of extra-curricular activities that girls are allowed to take part in increases, they will have less time and attention to spare for schoolwork. This would be a mechanism whereby more gender egalitarian values would have a negative effect on girls’ academic achievement, and hence a positive effect on the relative achievement of boys vs. girls.

We now turn to the influence of *peers.* An often mentioned factor behind boys’ underachievement in school is a social norm that putting effort into studying is regarded as “uncool” (Morris, 2012). This attitude is most common among boys, but it is also found among girls (Jackson and Nyström, 2015). An effect of gender egalitarian values could be that attitudes to studying become more similar between the genders, so that studying would be more “uncool” among girls in more gender egalitarian countries. As such negative attitudes to studying are likely to negatively affect achievement, this would amount to a positive effect of gender egalitarian values on the relative achievement of boys vs. girls.

Moreover, students’ achievement tends to be positively influenced by high expectations from their *teachers* (Jussim and Eccles, 1992). Teachers may hold various forms of gender bias in their expectations of students and in the various ways they interact with students (Frawley, 2005; Berekashvili, 2012). To the extent that teachers hold stereotypes that boys are rowdy and that girls are neat and disciplined, this could influence student achievement by lowering the relative achievement of boys vs. girls. More gender egalitarian values could reduce such gender stereotypes and thereby have a positive effect on the relative achievement of boys vs. girls.

Gender egalitarian values could also have a negative effect on the relative achievement of boys vs. girls. Specifically, parents with non-egalitarian attitudes about gender could have a lower interest in the future career of their girls than of their boys. Similarly, teachers with non-egalitarian attitudes about gender could think that girls do not need the highest levels of academic skills. If so, an increase in gender egalitarian values may lead to an increase in the encouragement that girls receive to excel in school (and hence a negative effect on the relative achievement of boys vs. girls).

#### Questions Addressed in the Present Research

The PISA and TIMSS assessments do not include measures of the gender egalitarian values of students’ family, friends, and teachers. We therefore cannot examine the specific pathways discussed above. Instead, we test the broader implication that country differences in gender egalitarian values may create country differences in the relative achievement of boys vs. girls. We do this by examining whether these variables are correlated at the country level.

In light of the lack of robustness of earlier findings on moderators of country differences in the relative achievement of boys vs. girls, we are especially interested in examining whether the moderating effect of gender egalitarian values is robust. We study robustness along the following dimensions.

##### Effects on gender differences vs. gender differences in effects

Our main question is whether gender egalitarian values are associated with gender differences in achievement. However, we are also interested in how gender egalitarian values are associated with boys’ and girls’ absolute levels of achievement. For instance, a positive effect on the relative achievement of boys vs. girls could arise either from a positive effect on boys’ achievement or from a negative effect on girls’ achievement.

##### Different sources of achievement scores

To explain that their analyses of 2003 PISA data and 2003 TIMSS data yielded inconsistent findings, Else-Quest et al. (2010) speculated that this might be due to the fact that PISA and TIMSS differ somewhat in the aim of the tests, in which case we should expect results from PISA and TIMSS data to be robustly different from each other. An alternative possibility is that the observed inconsistency was spurious, in which case we should expect findings from the two data sources to be generally consistent.

##### Mean vs. 90th percentile achievement scores

Although much research on gender differences in achievement focuses on the mean achievement levels of boys and girls, it is well-known that gender differences vary over the performance continuum (Robinson and Lubienski, 2011). Globally, the largest gender differences in mathematics performance tend to be found at the high end; by contrast, the high end in reading performance exhibits the smallest gender differences (Stoet and Geary, 2013). The high end of the performance continuum is of interest also because high achievers are particularly likely to enroll in higher education (Lubinski and Benbow, 2006). For these reasons, we will examine gender differences in achievement both at mean levels and at the 90th percentile. Measures of the mean achievement and 90th percentile achievement of boys and girls are provided by both PISA and TIMSS.

##### Different sources of gender egalitarian values scores

The concept of gender egalitarian values has been measured in various ways. As detailed in Section “Materials and Methods,” we shall use two different sources of data on how gender egalitarian values vary across countries: the World Values Survey (WVS) and the GLOBE project.

##### Controlling for other country-level variables

Whereas prior work has focused on gender equality in opportunities, the premise of the current work is that gender egalitarian values may exert a stronger influence on gender differences in achievement. Of course, gender egalitarian values and gender equality in opportunities are not independent of each other (Brandt, 2011). When analyzing the effect of the former variable, it is important to check that the results are robust to controlling for the latter variable. Further, both these aspects of gender equality are correlated with the standard of living in the country, which is in itself an important predictor of student achievement (Stoet and Geary, 2013). We therefore also control for measures of standard of living.

## Materials and Methods

Countries were included in this study if they satisfied three criteria: the country had participated at least once in PISA or TIMSS (excluding benchmark and off-grade participants), so that gender gaps in achievement could be calculated; gender equality measures for the country were available from the World Economic Forum, see below; data on gender egalitarian values in the country were available either from the WVS or the GLOBE project, see below. Supplementary Table S1 lists the 74 countries included in the study and indicates for which countries data were available from PISA, TIMSS, WVS, and GLOBE. The dataset can be accessed in the Open Science Framework data repository^2^.

### Girls’ and Boys’ Achievement Levels on PISA and TIMSS Tests Since 2000

PISA is an international assessment of 15-year-old students’ achievement in math, reading, and science (Schleicher et al., 2009). It is conducted by the Organization for Economic Co-operation and Development (OECD). PISA uses a representative sample of students from each participating country. Sample sizes are usually around 5000 per country but sometimes considerably larger. Data are available from six data collections: 2000, 2003, 2006, 2009, 2012, and 2015. Test scores are normalized, with a mean of 500 and a standard deviation of 100. Due to details of the design, comparability between different waves depends on the subject: whereas reading scores are comparable for all waves since 2000, math scores are only comparable from 2003 and onward, and science scores are only comparable from 2006 and onward. For these waves with comparable scores (six for reading, five for math, and four for science), the mean score and the score at the 90th percentile, calculated separately for boys and girls in each country, were downloaded from the National Center for Education Statistics^3^. Scores were obtained for 63 countries in our study. From the same source, we also downloaded the percentage of boys among participants in each country.

TIMSS is a similar international assessment of math and science achievement of students in the eighth grade (as well as the fourth grade, which is not used here), with most participants being about 14 years old. The assessment is conducted by the International Association for the Evaluation of Educational Achievement (IEA). Details on the design and execution of the TIMSS assessment are provided in reports from the IEA (e.g., Mullis et al., 2009). Although TIMSS differs from PISA in several ways, important similarities include that both use representative samples of similar sizes and that test scores are normalized in the same way. Thus, absolute levels and gender differences in country mean scores are roughly comparable between PISA and TIMSS. Since 2000, there have been four waves of TIMSS: 2003, 2007, 2011, and 2015. For these waves, the mean score and the score at the 90th percentile, calculated separately for girls and boys, were downloaded from the National Center for Education Statistics^3^. Data on scores and percentage of boys were obtained for 51 countries in our study.

### Gender Egalitarian Values

The WVS is a survey of human beliefs and values that has been conducted in six waves since 1981 by a global network of social scientists. Every wave is conducted over a period of 5 years and the sample of participating countries changes for every wave. Waves 3–6, conducted during 1994–2014, all included an index for gender egalitarian values called Equality (Welzel, 2013), which is based on three items:

*Jobs:* When jobs are scarce, men should have more right to a job than women.
*Politics:* On the whole, men make better political leaders than women do.
*University:* University is more important for a boy than for a girl.

The equality index is coded such that higher values of the index represent more egalitarian responses. We downloaded the full set of data from the WVS website^4^. We pooled waves 3–6 and calculated the country means of the equality index. WVS measures of gender egalitarian values were available for 67 countries in our study.

An alternative to the WVS is provided by the GLOBE project, which measured cultural practices and cultural values in societies across the world in the mid-1990s (House et al., 2004). For each participating country, the GLOBE project reported cultural values on nine dimensions, one of which is gender egalitarianism. The country index of gender egalitarian cultural values is based on survey responses to several attitude items on how society should be with respect to gender equality in education and leadership (e.g., “I believe that boys should be encouraged to attain a higher education more than girls,” “I believe that opportunities for leadership positions should be more available for men than for women”). The value of the composite index for each country was downloaded from the project website^5^. GLOBE measures of gender egalitarian values were available for 47 countries in our study. Data on separate items are not available.

### Control Variables

#### Gender Equality Indicators From the World Economic Forum

The Global Gender Gap Index (GGI) is a composite measure of gender equality published by the World Economic Forum every year since 2006. It has been used in many studies of the link between gender equality and math achievement (Guiso et al., 2008; Hyde and Mertz, 2009; Else-Quest et al., 2010; Stoet and Geary, 2013, 2015). The GGI is based on four component scores: “Economic participation and opportunity,” “Educational attainment,” “Health and survival,” and “Political empowerment.” All scores have a theoretical range from 0 to 1. Details on the GGI and its component scores are provided by the World Economic Forum (Hausmann et al., 2009). GGI scores for all available years were downloaded from https://tcdata360.worldbank.org.

#### The Human Development Index From the United Nations

The Human Development Index (HDI) is a composite measure of the standard of living in a country that is known to be a strong predictor of academic achievement levels (Stoet and Geary, 2013, 2015). The HDI is based on measures of people’s life expectancy at birth, education (expected and mean years of schooling), and income (GNI per capita). For details on the construction of the HDI and its component measures, see the report by the United Nations Development Program (UNDP, 2015). From their website^6^, we downloaded country scores of the HDI and its components for all years from 2000 and onward.

### Ethical Approval

No institutional ethical approval was necessary for carrying out this secondary data analysis of publicly available and fully anonymized datasets.

### Analysis

Our main analytic approach is to nest country-years in countries, using a two-level analysis (“mixed model”) on the form:

$$Y_{t\,c}=\beta_{0}+\beta_{1}\,G\,V_{c}+\beta_{2}\,H\,D\,I_{t\,c}+\beta_{3}\,G\,G\,I_{t\,c}+\beta_{4}\,P\,e\,r\,c\,B_{t\,c}+\beta_{5}\,Y\,r_{t}+u_{c}+e_{t\,c}$$

In the first set of analyses, the dependent variable denoted by *Y*_*tc*_ is the absolute achievement levels of boys and girls in country *c* in year *t*; in the second set of analyses, the dependent variable is instead the difference between these achievement levels (i.e., the relative achievement of boys vs. girls). Every predictor is centered at the global mean and scaled by standard deviation. The dependent variable is not standardized. We report unstandardized regression coefficients, which tell us effects in terms of the number of achievement score points by which the dependent variable increases when the predictor increases by one standard deviation.

The predictors are: the country’s gender egalitarian values (either WVS or GLOBE), denoted by *GV*_*c*_; the country’s prosperity in the given year^7^, denoted by *HDI*_*tc*_; the country’s level of gender equality in opportunities in the given year (or the closest year available^8^), denoted by *GGI*_*tc*_; the gender composition of students taking the test in the country in the given year (in terms of the percentage of boys in the sample), denoted by *PercB*_*tc*_; and *Yr*_*t*_ is the year of measurement. The country random intercept *u*_*c*_ and the error term *e*_*tc*_ are normally distributed with mean 0 and standard deviation $\sigma_{u}^{2}$ and $\sigma_{e}^{2}$.

Mixed model analyses were conducted in the lme4 package (Bates et al., 2014) in R, using restricted maximum likelihood (REML) estimation. Because the dependent variables are estimates of country levels of achievement based on random samples, we use an alternative model weighted by the inverse of the standard errors, normalized so that the sum of the weights is equal to the sample size. For each model, we estimate marginal pseudo R squared, which shows how much variance is explained by the fixed effects (Nakagawa et al., 2017). As some measures were not normally distributed, we estimate 95% confidence intervals based on 2.5 and 97.5 percentiles from 1000 bootstrap samples.

## Results

Descriptive statistics for all variables are reported in Table 1.

**TABLE 1:** Descriptive statistics.

**Assessment**	**Domain**	**Measure**	**Nobs**	**Nc**	**Nobs/Nc**	**Mean**	***SD***
PISA	Math	Boys, averages	244	63	3.87	469.9	54.98
		Boys, percentiles	244	63	3.87	588.96	58.06
		Relative achievement, averages	244	63	3.87	7.83	8.97
		Relative achievement, percentiles	244	63	3.87	16.13	9.31
		Girls, averages	244	63	3.87	462.07	53.01
		Girls, percentiles	244	63	3.87	572.83	55.85
	Reading	Boys, averages	279	63	4.43	447.42	52.13
		Boys, percentiles	279	63	4.43	569.43	51.97
		Relative achievement, averages	279	63	4.43	–36.46	12.67
		Relative achievement, percentiles	279	63	4.43	–25.53	10.21
		Girls, averages	279	63	4.43	483.88	51.14
		Girls, percentiles	279	63	4.43	594.96	50.66
	Science	Boys, averages	212	63	3.37	468.88	53.86
		Boys, percentiles	212	63	3.37	589.66	58.98
		Relative achievement, averages	212	63	3.37	–2.19	11.01
		Relative achievement, percentiles	212	63	3.37	7.11	10.49
		Girls, averages	212	63	3.37	471.06	50.48
		Girls, percentiles	212	63	3.37	582.54	55.19
TIMSS	Math	Boys, averages	134	51	2.63	464.83	71.18
		Boys, percentiles	134	51	2.63	576.15	67.48
		Relative achievement, averages	134	51	2.63	–1.76	11.13
		Relative achievement, percentiles	134	51	2.63	5.02	9.55
		Girls, averages	134	51	2.63	466.59	70.4
		Girls, percentiles	134	51	2.63	571.13	67.18
	Science	Boys, averages	134	51	2.63	474.25	68.03
		Boys, percentiles	134	51	2.63	585.19	57.32
		Relative achievement, averages	134	51	2.63	–1.65	17.34
		Relative achievement, percentiles	134	51	2.63	6.2	12.93
		Girls, averages	134	51	2.63	475.89	64
		Girls, percentiles	134	51	2.63	578.98	54.72
PISA		% Boys	280	63	4.44	0	1
TIMSS		% Boys	134	51	2.63	0	1
WVS			338	67	5.04	0	1
GLOBE			265	47	5.64	0	1
GGI			378	74	5.11	0	1
HDI			378	74	5.11	0	1
Year			378	74	5.11	0	1

*Nobs refers to the total number of observations (one observation for every year in which a country has participated in the assessment). Nc refers to the number of countries on which there are observations. Nobs/Nc is the average number of observations per country. Mean and *SD* refer to the mean and standard deviation of the observed scores.*

### Using Gender Egalitarian Values to Predict the Achievement Levels of Boys and Girls

Our first set of analyses predicted the mean and 90th percentile achievement scores of boys and girls from the gender egalitarian values in the country. Five separate cases of dependent variables were analyzed: achievement on the three PISA tests in reading, math, and science, as well as achievement on the two TIMSS tests in math and science. Moreover, each analysis was conducted two times using gender egalitarian values either from WVS or from GLOBE. Overall, the fixed effects explain around 55% of the observed variation in achievement levels. Most of the unexplained variation was on the country level (average SDc = 36.8) rather than on the country-year level (average SDres = 14.7).

**FIGURE 1:**
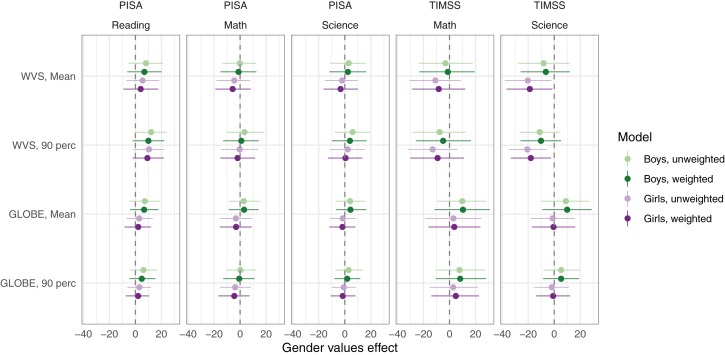
Estimated effects of gender egalitarian values on the mean and 90th percentile achievement of boys and girls. *Note*. The figure shows fixed effects of gender egalitarian values on the mean and 90th percentile of boys’ (green) and girls’ (violet) achievement, with bootstrapped 95% CI for weighted (dark) and unweighted (light) models. At the country level, all models included random intercepts. Entries estimate the number of points by which achievement scores increase when the gender egalitarian values increase by one standard deviation, holding all covariates constant. Different analyses are based on data from different samples of countries: 56 countries had both PISA and WVS data (total number of observations: *n* = 242 for reading, *n* = 213 for math, *n* = 186 for science), 40 countries had both PISA and GLOBE data (*n* = 203 for reading, *n* = 175 for math, *n* = 147 for science), 49 countries had both TIMSS and WVS data (*n* = 129), and 32 countries had both TIMSS and GLOBE data (*n* = 90). Standard deviations for the country random intercept ranged between 23.0 and 52.7 (mean = 36.8), and for residuals between 10.1 and 22.3 (mean = 14.7). The average marginal R squared was 0.55 (SD = 0.09, min = 0.38, max = 0.73).

The estimated effect of gender egalitarian values in each analysis is presented graphically in Figure 1; the exact numbers can be found in Supplementary Table S2. The first thing to note is that, with a single exception, none of the estimated effects of gender egalitarian values was significantly different from zero. Nonetheless, note the consistency in the difference between the estimated effects on boys’ and girls’ achievement. The gender difference in the estimated effect of gender egalitarian values consistently favored boys’ achievement. Averaging over all 20 analyses (using the weighted model), the estimated effect of gender egalitarian values on boys’ and girls’ achievement was 2.7 and −2.6, respectively, yielding a difference of 5.2 points. We briefly summarize the effects of the covariates by similarly calculating their average effects across the twenty analyses: HDI had a large positive effect on achievement, almost identical for boys and girls (41.0 vs. 41.1). The effects of the other covariates were smaller but still almost identical for boys and girls (GGI: 6.5 vs. 6.7; % boys in sample: 1.9 vs. 1.7; year: −8.3.1 vs. −7.9).

In sum, these analyses suggest two things. First, more gender egalitarian values may on the whole be less beneficial for girls’ achievement than for boys’ achievement. Second, although prosperity (HDI) and gender equality in opportunities (GGI) came out as more important determinants of countries’ achievement levels, on the whole these variables seem to be equally beneficial for girls’ and boys’ achievement. Our second set of analyses will provide further illumination of these patterns.

### Using Gender Egalitarian Values to Predict the Relative Achievement of Boys vs. Girls

**FIGURE 2:**
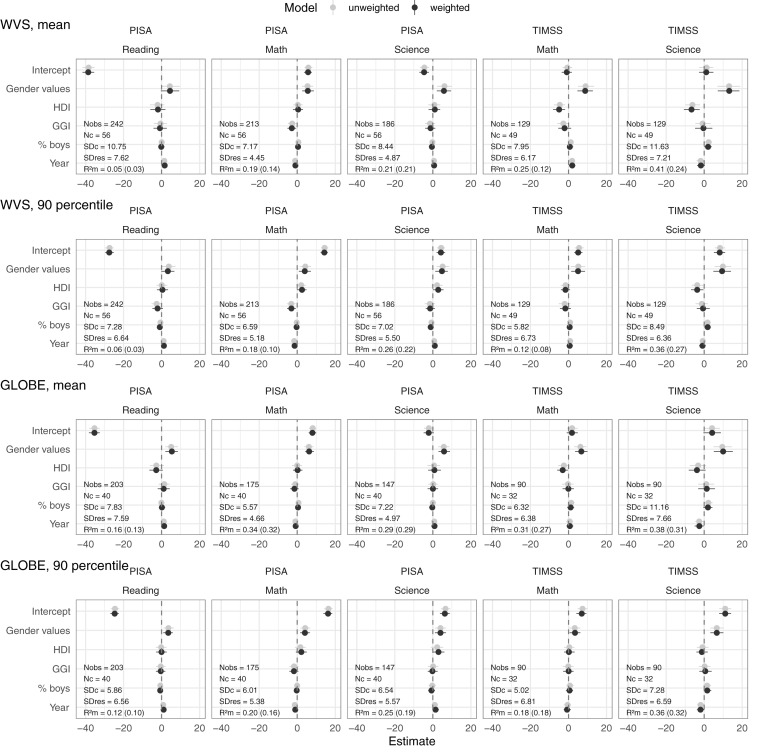
Mixed effects models of gender egalitarian values and covariates on the mean and 90th percentile relative achievement of boys vs. girls. *Note*. The figure shows fixed effects with 95% bootstrap CI for weighted (dark) and unweighted (light) models. For the weighted models, we display the standard deviations of the random country intercept (SDc) and of residuals (SDres), as well as marginal R-squared (R2m). Marginal R-squared for gender values alone is included in parentheses. All models included random intercepts at country level. All independent variables are centered on the mean and standardized to have unit standard deviation. Entries estimate by how many points the relative achievement of boys vs. girls tends to increase when the corresponding independent variable increases by one standard deviation and the other variables are held constant. Collinearity was not at problematic levels; all variance inflation factors were less than or equal to 3.3.

The above analyses of absolute achievement levels of boys and girls suggested that higher levels of gender egalitarian values may be associated with higher relative achievement of boys vs. girls. In the second set of analyses, we examined this relation directly, by using the relative achievement of boys vs. girls as the dependent variable. All fixed effect estimates are presented graphically in Figure 2; the exact numbers can be found in Supplementary Table S3.

To understand what Figure 2 is saying, first consider the intercepts. Because predictors are centered at their means, intercepts represent the estimated relative achievement of boys vs. girls when all predictors take their mean values. The figure shows substantial negative intercepts for reading, reflecting the global tendency for reading achievement to be lower among boys than among girls.

Our focus is the estimated effects of gender egalitarian values. Note that these were all positive and statistically significant. Averaged across all 20 analyses, an increase in the level of gender egalitarian values by one standard deviation was associated with an estimated increase of 6.0 points in the relative achievement of boys vs. girls. This finding dovetails nicely with our first set of analyses, which indicated that one standard deviation higher gender egalitarian values was on average 5.2 points more beneficial for boys than for girls.

Now consider the effects of the other predictors. Inspection of Figure 2 shows that, in every analysis, the effect of gender egalitarian values was larger than the effect of any other predictor. Moreover, the directions of the estimated effects of prosperity (HDI) and gender equality in opportunities (GGI) were inconsistent, sometimes positive and sometimes negative. The gender distribution of the sample was never a significant predictor. The estimated time trends had different signs in TIMSS and PISA for the same academic domains, suggesting that they lack reliability; we return to this issue in Section “Lack of Robustness of Estimates of How Gender Differences in Achievement Change Over Time.”

The fixed effects explain more substantial proportions of the variation in relative achievement in the domains of science (32% on average) and math (22% on average) than in the domain of reading (10% on average). As reported in Figure 2, most of the explained variance could be attributed to the effect of gender egalitarian values alone. The unexplained variation was roughly equally distributed between the country level (average SDc = 7.5) and the country-year level (average SDres = 6.1).

### Using Domain-Specific Covariates Instead of Indexes

In the above analyses of relative achievement, we used index measures of prosperity (HDI), and gender equality in opportunities (GGI) as covariates. These indexes are based on component measures from different domains. It is possible that domains matter, so that the use of indexes miss important aspects of what is actually going on. We therefore reran the analyses with the HDI and GGI indexes replaced by their domain-specific components (seven in total). The results were similar to the previous analyses. Across all 20 analyses, an increase in the level of gender egalitarian values by one standard deviation was associated with an estimated increase of ranging from 1.7 to 8.7 points (mean 4.4) in the relative mean achievement of boys vs. girls, whereas the covariates showed no robust effects. Thus, the effect of gender egalitarian values was not accounted for by any domain of prosperity or any domain of gender equality in opportunities.

### Which Are the Countries at Different Ends of Gender Egalitarian Values?

**FIGURE 3:**
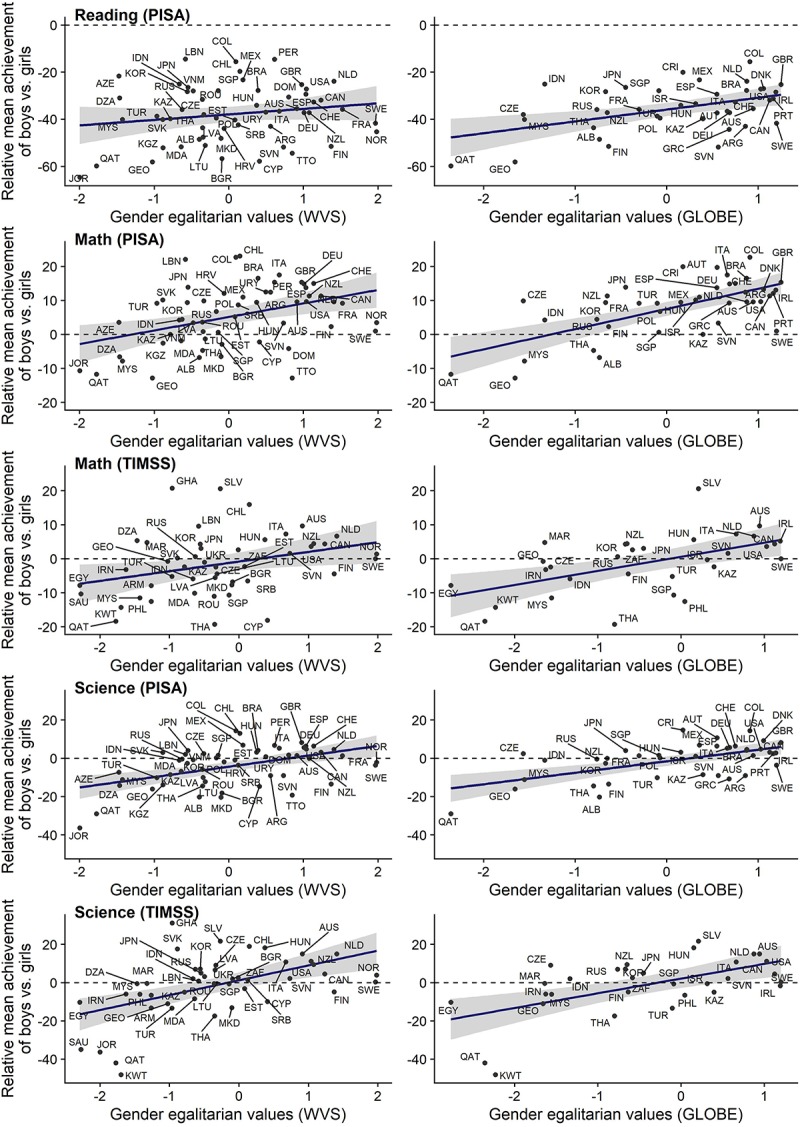
Scatterplots of the relative achievement of boys vs. girls per academic subject (reading, math, science) and assessment (PISA, TIMSS), averaged over all available waves and plotted against gender egalitarian values measured by WVS **(left)** or GLOBE **(right)**. The solid lines are the best fitting regression lines. The dashed lines indicate equal achievement of boys and girls; above the dashed line, the gender gap favors boys, below it, the gender gap favors girls.

The scatter plots in Figure 3 illustrate the relation between gender egalitarian values and the relative mean achievement of boys vs. girls without any controls. On the *y*-axis is simply the average of all available relative mean achievement scores for a country (per academic domain and assessment organization). On the *x*-axis is the country’s gender egalitarian values as measured by WVS (left) or GLOBE (right). Countries are identified by their three-letter country codes (ISO 3166-1 alpha-3, e.g., KWT for Kuwait). The scatter plots reveal culturally based clusters of countries. In the lower left corner, characterized by low levels of gender egalitarian values and low relative achievement of boys vs. girls, we tend to find countries on the Arabian Peninsula (Egypt, Qatar, Kuwait, and in the top panel also Jordan and Saudi Arabia). Whereas their economic conditions vary considerably, these neighboring countries are culturally similar. In the top right corner, characterized by high levels of gender egalitarian values and high relative achievement of boys vs. girls, we tend to find countries in Latin America, North America, Western and Middle Europe, Australia, and New Zealand. In between these groups, we tend to find countries from South-East Asia, Central Asia, and Eastern Europe.

The scatter plots for the domain of reading stand out from those of the other domains in three ways. First, there are fewer countries plotted. This is because reading is not included in TIMSS, so any country that has only participated in TIMSS will not have data on reading achievement. Second, all dots lie below the dashed reference line at zero in the scatter plots for reading, but not in the other scatter plots. Thus, we replicate the finding that girls consistently exhibit stronger achievement than boys in the domain of reading. Third, the points in the scatter plots for reading achievement are more widely scattered around the regression line than in the other scatter plots. This is consistent with our earlier observation that gender egalitarian values explain a smaller proportion of the total variation in the relative achievement of boys vs. girls in the reading domain than in the domains of mathematics and science.

### Variation in Results Across Different Waves of PISA

A previous study found that the relative achievement of boys vs. girls on PISA tests was somewhat lower in countries with more gender egalitarian values, as measured by WVS (Guiso et al., 2008). It is remarkable that we have here obtained a robust effect in the opposite direction. The explanation lies in that Guiso et al. only analyzed data from the 2003 wave of PISA. As discussed in Section “Introduction,” findings on gender differences in that dataset have failed to replicate in other waves of PISA (Stoet and Geary, 2013). To illustrate this phenomenon in the context of our study, we calculated the raw correlation between gender egalitarian values (WVS) and relative mean math achievement of boys vs. girls (PISA) separately for each wave (see Table 2). In each of the last four waves of PISA, we see a statistically significant positive raw correlation (ranging between 0.31 and 0.42), consistent with the results from our previous analysis of the pooled data. Moreover, consistent with the finding of Guiso et al. (mentioned above), the 2003 PISA data stands out by yielding a negative correlation (−0.17). Thus, the finding for 2003 does not depend on the details of how the data analysis was conducted, which differed somewhat between our study and the study of Guiso et al.^9^ Instead, the crucial difference between the studies is that we pooled data from numerous waves to obtain a more representative dataset. The rest of Table 2 shows that correlations vary substantially across waves also for the PISA reading test and the TIMSS math test.

**TABLE 2:** Correlations between gender egalitarian values (WVS) and relative mean achievement of boys vs. girls for each wave of PISA and TIMSS.

**PISA**				**TIMSS**			
**Domain**	**Year**	***r***	**95% CI**	***n***	***r***	**95% CI**	***n***
Reading	2000	–0.06	[−0.37, 0.29]	30			
Reading	2003	–0.40	[−0.70, −0.01]	27			
Reading	2006	0.20	[−0.17, 0.53]	41			
Reading	2009	0.20	[−0.06, 0.46]	47			
Reading	2012	0.19	[−0.19, 0.49]	47			
Reading	2015	0.27	[−0.04, 0.54]	50			
Math	2003	–0.17	[−0.56, 0.23]	27	0.23	[−0.14, 0.56]	35
Math	2006	0.42	[0.10, 0.65]	42			
Math	2007				0.36	[0.08, 0.59]	35
Math	2009	0.41	[0.16, 0.64]	47			
Math	2011				0.40	[0.08, 0.68]	31
Math	2012	0.40	[0.05, 0.66]	47			
Math	2015	0.31	[0.02, 0.58]	50	0.59	[0.40, 0.78]	28
Science	2003				0.43	[0.11, 0.68]	35
Science	2006	0.45	[0.07, 0.68]	42			
Science	2007				0.55	[0.30, 0.73]	35
Science	2009	0.53	[0.29, 0.72]	47			
Science	2011				0.58	[0.28, 0.82]	31
Science	2012	0.45	[0.05, 0.68]	47			
Science	2015	0.43	[0.12, 0.65]	50	0.68	[0.54, 0.81]	28

*The first two columns for each assessment (PISA, TIMSS) report Pearson correlations with bootstrapped 95% confidence intervals. The last column reports the sample size, that is, the number of countries in our dataset that participated in the assessment in the given year.*

### Lack of Robustness of Estimates of How Gender Differences in Achievement Change Over Time

As a supplementary analysis, we examined whether we can reliably estimate for each country how gender differences in achievement change over time. To examine this question, we calculated separate change estimates from TIMSS data and PISA data for math and science achievement. To obtain change estimates for a given country, we used the relative achievement of boys vs. girls in each wave as data points and performed a linear regression on the year of the wave; the resulting regression coefficient is an estimate of the direction and rate by which the relative achievement of boys vs. girls has changed in that country. In this way, separate change estimates for TIMSS and PISA were obtained for all 23 countries in which data has to be available from at least two waves of each assessment). These change estimates reflect both genuine change and noise from sampling errors. If the signal from genuine change dominates, we would expect change estimates in the same domain from the two assessments to be strongly positively correlated. However, they were not; correlations were close to zero both in the mathematics domain, *r* = −0.09, *p* = 0.66, and in the science domain, *r* = 0.18, *p* = 0.42. We conclude that the data are not sufficient to yield reliable estimates of how the relative achievement of boys vs. girls changes over time in different countries.

## Discussion

The present research examined the relative achievement of boys vs. girls on standardized tests and how it varies with societies’ attitudes toward gender equality. A clear pattern emerged. Across all assessed domains (reading, mathematics, science), the relative achievement of boys vs. girls was higher in countries with high levels of gender egalitarian values. These findings are based on data from several waves of the PISA and TIMSS assessments, together covering 74 countries. The same findings were obtained regardless of whether gender differences in achievement were measured using PISA or TIMSS and whether measured at the mean or 90th percentile, and regardless of whether gender egalitarian values were measured by WVS or GLOBE. Thus, the findings appear robust.

It is noteworthy that the findings of a previous study (Guiso et al., 2008), which only used data from the 2003 wave of PISA, did not replicate in our larger dataset. As discussed earlier, results from a single wave may be unreliable due to sampling errors and limited sets of participating countries.

Whereas the same relationship between gender egalitarian values and the relative achievement of boys vs. girls was found across different academic domains, it is important to note that the baseline level of the relative achievement of boys vs. girls varies greatly between reading on the one hand and mathematics and science on the other hand. With respect to reading, boys underachieve relative to girls in every country in our sample. This contrasts with the results in math and science, where underachievement of boys is found mainly in countries with low levels of gender egalitarian values. In countries with high levels of gender egalitarian values, boys tend to overachieve relative to girls on these math and science tests. Note, however, that even in countries where boys achieve better than girls on math and science tests, they do not necessarily do better in school on these subjects, because school grades also reflect other aspects, such as self-discipline, which seem to favor girls (Duckworth and Seligman, 2006).

### What Explains the Association Between Gender Egalitarian Values and Gender Differences in Achievement?

Future research can test possible explanations of the observed association between gender egalitarian values and gender differences in academic achievement. It would be ideal if future PISA and TIMSS questionnaires were to include items bearing on the gender egalitarian values of the friends, teachers, and parents of individual students, to allow examination of the mechanisms suggested in Section “Introduction.” The common thread of those hypothetical mechanisms is that gender egalitarian values could have the (unintended) consequence that girls become less engaged with school. Note that this is consistent with the results in Section “Using Gender Egalitarian Values to Predict the Achievement Levels of Boys and Girls,” which (although not statistically significant) indicated that the independent effect of more gender egalitarian values on girls’ absolute achievement levels is slightly negative. In this context, it is worth considering how our results can be reconciled with results from studies of the relative achievement of boys vs. girls among second-generation immigrants, in which higher levels of gender equality in the country of ancestry were associated with *lower* relative achievement of boys vs. girls (Nollenberger et al., 2016; Rodríguez-Planas and Nollenberger, 2018). Assuming that the gender egalitarian values of immigrants tend to reflect the level of gender equality in the country they come from, this finding seems to contrast with our finding that more gender egalitarian values is associated with *higher* relative achievement of boys vs. girls. We suggest that the difference may be explained by the specific situation of second-generation immigrants, for whom the values of their parents may conflict with the values of teachers and friends in the new country, which could lead to less compliance with parental values (Choi et al., 2008).

### Causality

We now consider the question of causal direction. Could it be that the causal direction is the reverse to what we have assumed above, so that gender differences in achievement would affect cultural values? We find this direction implausible. In order for gender differences in achievement to be able to influence cultural values, a minimum requirement should be that people can readily observe these gender differences at a sufficient level of accuracy to distinguish between different countries. But we know that gender differences in achievement are much too small for that; indeed, we needed this statistical analysis of millions of students to be able to quantify them with sufficient accuracy.

Yet another possibility to consider is that the observed association could follow from some third variable driving both cultural values and achievement levels. We have tried to account for this in our analyses by controlling for various indicators of prosperity and gender equality in opportunities, which prior research would suggest to be the most likely candidates. We cannot exclude that there may be other important unobserved country-level factors. However, in the absence of any theory about such factors, we tentatively conclude that more gender egalitarian values in a country may in fact be a cause of higher relative achievement of boys vs. girls.

To establish causality, it would have been ideal to examine whether changes over time in values predict changes over time in gender differences in achievement. Unfortunately, we found that such an examination is not meaningful due to the data being insufficient to reliably measure change over time.

### Effect Sizes of Gender Differences

Here we have examined gender differences in achievement in terms of differences in test scores. Much previous research has instead examined gender differences in terms of Cohen’s *d*, which is the gender difference in test scores divided by the standard deviation in test scores in the country. These measures are extremely closely correlated (typically *r* > 0.99). Approximate quantitative results for gender differences in terms of Cohen’s *d* are easily obtained by a simple rule of thumb: divide results for raw score differences by 100, the typical standard deviation. For instance, our analyses indicated that on average an increase of gender egalitarian values by one standard deviation corresponds to an increase by about 6 points in the relative achievement of boys vs. girls, which means a change by 0.06 in Cohen’s *d*. A change in gender egalitarian values from the very low level on the Arabian Peninsula to the very high level in Western Europe amounts to a change in Cohen’s *d* on the order of 0.3. Thus, the variation between countries in gender differences in achievement is not negligible, but gender differences are on the whole quite small compared to the variation in achievement between students in the same country.

### Unexplained Variation

Finally, we emphasize that there is still a lot of unexplained variation in gender differences in achievement. Figure 1 shows that the relative math achievement of boys vs. girls in Ghana was much higher than expected from gender egalitarian values alone, whereas Sweden, Norway, Cyprus, Trinidad and Tobago, and Dominican Republic are outliers in the opposite direction. Future research could address these outliers. For instance, it would be interesting to understand why the latter two Caribbean countries differ so much from neighboring Latin American countries with respect to gender differences in achievement, given that they have similar levels of gender egalitarian values.

## Conclusion

The main conclusion of our study of 74 countries is that gender egalitarian values seem to play a role in shaping gender differences in academic achievement that has not been documented in previous research. A prior study of 2003 PISA data found that the relative mathematics achievement of boys vs. girls tended to be lower in countries with more gender egalitarian values. In stark contrast to this finding, our analysis of a much larger dataset found that, regardless of academic domain, the relative achievement of boys vs. girls tended to be *higher* in countries with more gender egalitarian values. By comparison, measures of gender equality in opportunities had no clear independent effect on gender differences in academic achievement. Cultural values are pervasive and could influence almost every aspect of the academic environment of boys and girls: family, friends, and teachers. The exact pathway by which gender egalitarian values influence the academic achievement of boys and girls is still an open question, but plausible candidates include their freedom to engage in extracurricular activities and expectations on their academic efforts.

## Data Availability Statement

The dataset analyzed for this study can be found in the OSF https://osf.io/v7bqt/.

## Ethics Statement

Ethical review and approval was not required for the study on human participants in accordance with the local legislation and institutional requirements. Written informed consent from the participants’ legal guardian/next of kin was not required to participate in this study in accordance with the national legislation and the institutional requirements.

## Author Contributions

KE conceived of the study, performed the statistical analysis, and wrote the manuscript. MB contributed to the literature review and provided critical input on the manuscript. All authors read and approved the submitted version.

## Conflict of Interest

The authors declare that the research was conducted in the absence of any commercial or financial relationships that could be construed as a potential conflict of interest.

## Footnotes

1 nces.ed.gov

2 https://osf.io/v7bqt/

3 nces.ed.gov

4 worldvaluessurvey.org/wvs.jsp

5 globeproject.com

6 hdr.undp.org

7 Lebanon is missing information for the education subindex of the HDI for the years 2000 and 2003; we used the value from 2005 to reestimate the HDI index for those years.

8 GGI are generally available from 2006, so for 2000 and 2003, it is the 2006 data that are used. For some countries, however, GGI data start later than 2006; for earlier years, we then use data from the earliest year available, which was 2007 for Azerbaijan, Armenia, Vietnam, and Qatar, 2010 for Lebanon, and 2012 for Serbia.

9 For instance, out of concern about possible differential drop-out rates across genders, Guiso et al. excluded all students in the lower half of the distribution of socio-economic status where drop-out rates were assumed to be higher. By comparison, we used all students and instead included the gender composition as a covariate.
