# Supplementary material for: The Relation Between Gender Egalitarian Values and Gender Differences in Academic Achievement
Source: Front Psychol. 2020 Feb 20;11:236. doi: 10.3389/fpsyg.2020.00236 (PMC7044344; doi:10.3389/fpsyg.2020.00236)
Supplement: Supplementary file 1 [file Table_1.DOCX]

The Relation between Gender Egalitarian Values and Gender Differences in Academic Achievement

# Supplemental Material

Table S1

*Participating Countries in PISA, TIMSS, WVS, and GLOBE*

| Country | PISA | TIMSS | WVS | GLOBE |
| --- | --- | --- | --- | --- |
| Albania | X | - | X | X |
| Algeria | X | X | X | - |
| Argentina | X | - | X | X |
| Armenia | - | X | X | - |
| Australia | X | X | X | X |
| Austria | X | - | - | X |
| Azerbaijan | X | - | X | - |
| Brazil | X | - | X | X |
| Bulgaria | X | X | X | - |
| Canada | X | X | X | X |
| Chile | X | X | X | - |
| Colombia | X | X | X | X |
| Costa Rica | X | - | - | X |
| Croatia | X | - | X | - |
| Cyprus | X | X | X | - |
| Czech Rep. | X | X | X | X |
| Denmark | X | - | - | X |
| Dominican Rep. | X | - | X | - |
| Egypt | - | X | X | X |
| El Salvador | - | X | X | X |
| Estonia | X | X | X | - |
| Finland | X | X | X | X |
| France | X | - | X | X |
| Georgia | X | X | X | X |
| Germany | X | - | X | X |
| Ghana | - | X | X | - |
| Greece | X | - | - | X |
| Hungary | X | X | X | X |
| Indonesia | X | X | X | X |
| Iran | - | X | X | X |
| Ireland | X | X | - | X |
| Israel | X | X | - | X |
| Italy | X | X | X | X |
| Japan | X | X | X | X |
| Jordan | X | X | X | - |
| Kazakhstan | X | X | X | X |
| Korea | X | X | X | X |
| Kuwait | - | X | X | X |
| Kyrgyzstan | X | - | X | - |
| Latvia | X | X | X | - |
| Lebanon | X | X | X | - |
| Lithuania | X | X | X | - |
| Macedonia | X | X | X | - |
| Malaysia | X | X | X | X |
| Mexico | X | - | X | X |
| Moldova | X | X | X | - |
| Morocco | - | X | X | X |
| Netherlands | X | X | X | X |
| New Zealand | X | X | X | X |
| Norway | X | X | X | - |
| Peru | X | - | X | - |
| Philippines | - | X | X | X |
| Poland | X | - | X | X |
| Portugal | X | - | - | X |
| Qatar | X | X | X | X |
| Romania | X | X | X | - |
| Russia | X | X | X | X |
| Saudi Arabia | - | X | X | - |
| Serbia | X | X | X | - |
| Singapore | X | X | X | X |
| Slovakia | X | X | X | - |
| Slovenia | X | X | X | X |
| South Africa | - | X | X | X |
| Spain | X | - | X | X |
| Sweden | X | X | X | X |
| Switzerland | X | - | X | X |
| Thailand | X | X | X | X |
| Trinidad Tobago | X | - | X | - |
| Turkey | X | X | X | X |
| Ukraine | - | X | X | - |
| United Kingdom | X | - | X | X |
| United States | X | X | X | X |
| Uruguay | X | - | X | - |
| Vietnam | X | - | X | - |

*Note*. An X means that data from that source exist for that country for the time period we study. The data can be accessed in the Open Science Framework data repository (<https://osf.io/v7bqt/>).

| Table S2  Point estimates and 95% bootstrap CI for estimated effects of gender egalitarian values measured by WVS and GLOBE on the mean and 90^th^ percentile achievement of boys and girls, presented graphically in Figure 1 in the main text. | | | | | |
| --- | --- | --- | --- | --- | --- |
| Assessment: | PISA | | | TIMSS | |
| Domain: | Reading | Math | Science | Math | Science |
| *Gender egalitarian values measured by WVS, Mean achievement* | | | | | |
| Effect on boys |  |  |  |  |  |
| Unweighted | 8.0 [-5.3, 21.2] | -0.3 [-13.7, 12.3] | 2.9 [-11.3, 16.2] | -3.2 [-23.6, 17.9] | -8.1 [-27.8, 11.7] |
| Weighted | 6.9 [-6.3, 20.0] | -1.2 [-15.1, 12.5] | 2.3 [-11.9, 16.5] | -1.3 [-23.2, 19.3] | -6.5 [-25.8, 12.0] |
| Effect on girls |  |  |  |  |  |
| Unweighted | 5.6 [-6.9, 18.2] | -4.5 [-18.1, 7.7] | -2.1 [-15.5, 9.9] | -10.8 [-30.7, 8.6] | -20.3 [-37.9, -2.2] |
| Weighted | 4.0 [-9.2, 17.6] | -5.7 [-19.0, 8.1] | -3.3 [-16.4, 10.1] | -8.3 [-28.6, 12.1] | -18.7 [-36.6, -1.4] |
| *Gender egalitarian values measured by GLOBE, Mean achievement* | | | | | |
| Effect on boys |  |  |  |  |  |
| Unweighted | 7.3 [-4.9, 18.8] | 2.9 [-8.6, 15.4] | 4.1 [-7.1, 15.5] | 9.8 [-9.2, 28.6] | 9.0 [-9.7, 26.8] |
| Weighted | 6.7 [-4.1, 17.7] | 3.0 [-8.7, 14.3] | 4.4 [-6.9, 16.6] | 10.3 [-11.6, 30.8] | 9.9 [-9.1, 28.7] |
| Effect on girls |  |  |  |  |  |
| Unweighted | 2.9 [-6.8, 13.3] | -3.2 [-15.2, 8.8] | -1.6 [-11.2, 8.7] | 3.0 [-19.0, 24.1] | -1.1 [-18.0, 16.0] |
| Weighted | 2.1 [-8.4, 12.0] | -3.1 [-15.6, 8.9] | -1.8 [-11.9, 8.2] | 3.6 [-16.2, 23.7] | -0.5 [-17.0, 16.2] |
| *Gender egalitarian values measured by WVS, 90^th^ percentile achievement* | | | | | |
| Effect on boys |  |  |  |  |  |
| Unweighted | 12.0 [-0.6, 24.0] | 3.3 [-10.0, 18.7] | 6.0 [-7.9, 19.4] | -7.5 [-28.5, 12.2] | -11.1 [-26.7, 4.6] |
| Weighted | 10.0 [-2.2, 22.3] | 1.0 [-13.0, 14.4] | 3.9 [-9.8, 16.8] | -4.9 [-25.8, 16.5] | -10.2 [-25.9, 5.2] |
| Effect on girls |  |  |  |  |  |
| Unweighted | 10.4 [-1.4, 22.1] | -0.3 [-14.6, 13.7] | 2.3 [-11.3, 15.3] | -13.0 [-32.1, 6.2] | -20.6 [-34.8, -5.7] |
| Weighted | 9.2 [-2.1, 21.8] | -1.9 [-15.2, 11.5] | 0.5 [-13.0, 13.5] | -9.1 [-30.1, 11.1] | -18.0 [-33.3, -2.4] |
| *Gender egalitarian values measured by GLOBE, 90^th^ percentile achievement* | | | | | |
| Effect on boys |  |  |  |  |  |
| Unweighted | 6.1 [-4.4, 16.7] | 0.2 [-10.9, 12.5] | 2.9 [-7.3, 14.0] | 7.6 [-10.7, 27.2] | 5.3 [-8.4, 19.2] |
| Weighted | 4.9 [-4.7, 15.4] | -0.7 [-12.9, 11.0] | 1.9 [-8.0, 11.9] | 8.2 [-10.5, 28.1] | 5.3 [-8.6, 19.0] |
| Effect on girls |  |  |  |  |  |
| Unweighted | 3.0 [-6.2, 12.1] | -3.7 [-15.3, 8.4] | -0.9 [-10.1, 8.4] | 2.8 [-15.1, 21.7] | -1.9 [-15.4, 11.3] |
| Weighted | 2.0 [-7.4, 10.6] | -4.5 [-16.8, 7.2] | -1.6 [-10.9, 8.2] | 4.8 [-14.0, 22.7] | -0.8 [-14.1, 12.2] |
|  | | | | | |

| Table S3  Point estimates and 95% bootstrap CI for mixed effects models of gender egalitarian values and covariates of the mean and 90th percentile achievement of boys vs. girls, presented graphically in Figure 2 in the main text. | | | | | | | |
| --- | --- | --- | --- | --- | --- | --- | --- |
| Assessment: | PISA | | | | TIMSS | | |
| Domain: | Reading | Math | Science | Math | | Science |  |
| *Gender egalitarian values measured by WVS, Mean achievement* | | | | | | | |
| Intercept |  |  |  |  | |  |  |
| Unweighted | -38.4 [-41.5, -35.3] | 5.9 [3.9, 8.0] | -4.5 [-6.8, -2.0] | -0.6 [-3.1, 2.0] | | 1.3 [-2.6, 4.8] |  |
| Weighted | -38.6 [-41.6, -35.5] | 5.7 [3.6, 7.7] | -4.6 [-7.1, -2.1] | -0.9 [-3.6, 1.7] | | 1.1 [-2.6, 4.8] |  |
| Gender values |  |  |  |  | |  |  |
| Unweighted | 4.3 [-0.2, 9.5] | 5.4 [2.5, 8.4] | 6.1 [2.3, 9.9] | 9.0 [5.0, 13.4] | | 13.1 [7.0, 19.0] |  |
| Weighted | 4.4 [-0.4, 9.3] | 5.6 [2.6, 8.7] | 5.8 [2.1, 9.5] | 8.8 [4.7, 12.9] | | 13.2 [7.2, 18.5] |  |
| HDI |  |  |  |  | |  |  |
| Unweighted | -2.2 [-6.1, 1.5] | 0.3 [-2.2, 2.9] | 0.8 [-2.5, 3.9] | -4.6 [-7.7, -1.8] | | -6.1 [-9.8, -2.2] |  |
| Weighted | -1.9 [-5.9, 2.0] | 0.5 [-2.2, 3.0] | 1.0 [-2.3, 3.8] | -4.9 [-8.1, -1.8] | | -6.6 [-10.6, -2.4] |  |
| GGI |  |  |  |  | |  |  |
| Unweighted | -0.6 [-4.1, 2.8] | -2.6 [-4.9, -0.3] | -1.5 [-4.1, 1.1] | -2.7 [-5.9, 0.3] | | -0.8 [-4.7, 3.4] |  |
| Weighted | -0.8 [-4.3, 2.7] | -2.9 [-5.2, -0.6] | -1.2 [-4.0, 1.3] | -2.1 [-5.3, 1.4] | | -0.4 [-4.8, 4.3] |  |
| % boys |  |  |  |  | |  |  |
| Unweighted | 0.0 [-1.6, 1.7] | 0.6 [-0.5, 1.7] | -0.2 [-1.6, 1.2] | 1.2 [-0.3, 2.8] | | 2.3 [0.6, 4.2] |  |
| Weighted | -0.2 [-1.8, 1.5] | 0.4 [-0.6, 1.5] | -0.5 [-1.9, 0.8] | 0.7 [-0.8, 2.4] | | 2.0 [0.0, 4.0] |  |
| Year |  |  |  |  | |  |  |
| Unweighted | 1.4 [-0.0, 2.9] | -1.1 [-2.2, -0.0] | 0.6 [-0.8, 2.0] | 1.9 [0.2, 3.5] | | -1.9 [-4.0, 0.2] |  |
| Weighted | 1.7 [0.0, 3.2] | -1.0 [-2.0, 0.1] | 0.6 [-0.8, 2.2] | 1.9 [0.3, 3.6] | | -1.7 [-3.7, 0.4] |  |
|  | | | | | | | |
| *Gender egalitarian values measured by WVS, 90^th^ percentile achievement* | | | | | | | |
| Intercept |  |  |  |  | |  |  |
| Unweighted | -27.4 [-29.7, -25.3] | 14.4 [12.5, 16.4] | 4.4 [2.2, 6.4] | 5.6 [3.2, 8.0] | | 8.3 [5.5, 11.1] |  |
| Weighted | -27.5 [-29.6, -25.2] | 14.2 [12.3, 16.1] | 4.2 [1.9, 6.4] | 5.1 [3.0, 7.3] | | 8.2 [5.2, 11.0] |  |
| Gender values |  |  |  |  | |  |  |
| Unweighted | 3.8 [0.2, 7.3] | 4.2 [1.2, 7.2] | 5.1 [1.9, 8.6] | 5.1 [1.3, 8.9] | | 9.9 [5.5, 14.2] |  |
| Weighted | 3.3 [-0.2, 6.7] | 4.0 [0.9, 7.2] | 4.7 [1.3, 7.8] | 5.0 [1.4, 8.7] | | 9.5 [4.8, 14.1] |  |
| HDI |  |  |  |  | |  |  |
| Unweighted | 0.1 [-2.7, 3.0] | 2.1 [-0.4, 4.6] | 2.2 [-0.7, 5.1] | -1.5 [-4.0, 1.0] | | -3.6 [-6.6, -0.6] |  |
| Weighted | 0.5 [-2.4, 3.4] | 2.5 [-0.2, 4.9] | 2.8 [0.1, 5.6] | -1.6 [-4.0, 0.8] | | -3.6 [-6.9, -0.4] |  |
| GGI |  |  |  |  | |  |  |
| Unweighted | -2.6 [-5.3, 0.2] | -3.2 [-5.4, -0.8] | -1.6 [-4.3, 1.1] | -2.0 [-5.2, 1.1] | | -1.2 [-4.5, 1.9] |  |
| Weighted | -2.1 [-4.9, 0.7] | -3.1 [-5.4, -0.8] | -1.4 [-3.7, 1.1] | -1.7 [-4.5, 1.3] | | -0.6 [-3.8, 2.9] |  |
| % boys |  |  |  |  | |  |  |
| Unweighted | -0.6 [-2.0, 0.7] | -0.2 [-1.4, 1.1] | -0.7 [-2.3, 0.8] | 0.8 [-0.8, 2.3] | | 1.8 [0.0, 3.5] |  |
| Weighted | -0.9 [-2.3, 0.6] | -0.4 [-1.6, 0.8] | -1.2 [-2.7, 0.2] | 0.7 [-1.0, 2.1] | | 1.8 [0.1, 3.5] |  |
| Year |  |  |  |  | |  |  |
| Unweighted | 1.1 [-0.1, 2.2] | -1.6 [-2.7, -0.4] | 0.7 [-0.9, 2.2] | 0.8 [-0.9, 2.5] | | -0.7 [-2.4, 1.0] |  |
| Weighted | 1.2 [0.1, 2.3] | -1.4 [-2.5, -0.3] | 1.0 [-0.4, 2.5] | 0.6 [-1.0, 2.3] | | -0.9 [-2.7, 0.8] |  |
|  | | | | | | | |
| *Gender egalitarian values measured by GLOBE, Mean achievement* | | | | | | | |
| Intercept |  |  |  |  | |  |  |
| Unweighted | -35.3 [-38.2, -32.5] | 8.2 [6.2, 10.2] | -2.1 [-4.6, 0.6] | 1.8 [-0.8, 4.8] | | 4.1 [-0.3, 8.3] |  |
| Weighted | -35.3 [-38.2, -32.6] | 8.0 [6.0, 10.0] | -2.2 [-4.8, 0.5] | 1.8 [-0.9, 4.8] | | 4.2 [-0.4, 8.8] |  |
| Gender values |  |  |  |  | |  |  |
| Unweighted | 5.0 [2.0, 8.6] | 6.1 [3.7, 8.6] | 5.8 [2.8, 8.6] | 6.3 [2.8, 9.5] | | 9.6 [5.0, 14.5] |  |
| Weighted | 5.4 [2.0, 8.6] | 6.3 [4.2, 8.8] | 5.9 [3.0, 9.0] | 6.7 [3.5, 10.0] | | 10.0 [5.1, 15.0] |  |
| HDI |  |  |  |  | |  |  |
| Unweighted | -2.8 [-6.2, 1.2] | -0.0 [-2.7, 2.4] | 0.6 [-2.9, 3.8] | -2.4 [-5.4, 0.5] | | -3.2 [-7.6, 1.1] |  |
| Weighted | -2.8 [-6.5, 0.6] | 0.2 [-2.2, 2.7] | 0.8 [-2.5, 4.2] | -3.0 [-5.9, -0.3] | | -3.8 [-8.1, 0.8] |  |
| GGI |  |  |  |  | |  |  |
| Unweighted | 1.4 [-1.6, 4.3] | -1.0 [-3.3, 1.1] | 0.1 [-2.4, 2.8] | -0.3 [-3.5, 2.7] | | 1.1 [-3.1, 5.2] |  |
| Weighted | 1.1 [-2.0, 4.2] | -1.4 [-3.7, 0.7] | 0.0 [-2.8, 2.7] | -0.0 [-3.1, 2.8] | | 1.4 [-3.1, 5.6] |  |
| % boys |  |  |  |  | |  |  |
| Unweighted | -0.1 [-1.7, 1.6] | 0.7 [-0.6, 2.0] | -0.0 [-1.6, 1.6] | 1.4 [-0.4, 3.2] | | 2.2 [-0.1, 4.5] |  |
| Weighted | 0.2 [-1.4, 1.8] | 0.4 [-0.8, 1.6] | -0.3 [-1.9, 1.2] | 1.1 [-0.6, 2.9] | | 2.0 [-0.4, 4.3] |  |
| Year |  |  |  |  | |  |  |
| Unweighted | 1.2 [-0.2, 2.5] | -1.0 [-2.1, 0.1] | 0.7 [-0.8, 2.3] | 0.6 [-1.2, 2.4] | | -2.6 [-4.7, -0.4] |  |
| Weighted | 1.4 [-0.1, 2.8] | -0.8 [-1.9, 0.3] | 0.8 [-0.8, 2.3] | 0.7 [-1.1, 2.5] | | -2.6 [-5.0, -0.3] |  |
|  | | | | | | | |
| *Gender egalitarian values measured by WVS, 90^th^ percentile achievement* | | | | | | | |
| Intercept |  |  |  |  | |  |  |
| Unweighted | -24.5 [-26.7, -22.3] | 16.5 [14.2, 18.7] | 6.7 [4.3, 9.2] | 7.4 [4.8, 10.0] | | 11.1 [7.9, 14.2] |  |
| Weighted | -24.7 [-26.9, -22.5] | 16.2 [13.8, 18.4] | 6.3 [3.7, 8.8] | 7.0 [4.2, 9.4] | | 11.1 [7.9, 14.2] |  |
| Gender values |  |  |  |  | |  |  |
| Unweighted | 3.6 [1.0, 6.4] | 4.2 [1.7, 6.8] | 4.0 [1.1, 6.8] | 3.2 [0.2, 6.2] | | 6.7 [3.2, 10.2] |  |
| Weighted | 3.5 [0.9, 5.9] | 4.1 [1.5, 6.6] | 4.0 [1.1, 6.9] | 3.4 [0.5, 6.2] | | 6.7 [3.3, 10.1] |  |
| HDI |  |  |  |  | |  |  |
| Unweighted | -0.3 [-3.0, 2.6] | 1.7 [-1.2, 4.5] | 2.3 [-1.0, 5.4] | 0.4 [-2.3, 3.1] | | -0.9 [-3.9, 2.3] |  |
| Weighted | -0.1 [-2.9, 2.7] | 2.1 [-0.7, 5.0] | 3.0 [0.0, 6.0] | 0.2 [-2.3, 3.2] | | -1.2 [-4.0, 1.9] |  |
| GGI |  |  |  |  | |  |  |
| Unweighted | -0.4 [-2.9, 2.1] | -1.6 [-4.1, 0.7] | 0.1 [-2.6, 2.9] | -0.2 [-3.0, 2.7] | | 0.3 [-2.8, 3.3] |  |
| Weighted | -0.5 [-2.9, 2.0] | -1.8 [-4.2, 0.6] | -0.2 [-2.7, 2.5] | 0.0 [-2.8, 2.6] | | 0.6 [-2.5, 3.9] |  |
| % boys |  |  |  |  | |  |  |
| Unweighted | -0.6 [-2.0, 0.8] | -0.1 [-1.5, 1.5] | -0.3 [-2.2, 1.6] | 0.7 [-1.1, 2.6] | | 1.6 [-0.5, 3.6] |  |
| Weighted | -0.7 [-2.1, 0.6] | -0.3 [-1.5, 1.1] | -0.7 [-2.4, 1.1] | 0.5 [-1.3, 2.4] | | 1.7 [-0.2, 3.7] |  |
| Year |  |  |  |  | |  |  |
| Unweighted | 0.9 [-0.3, 2.1] | -1.3 [-2.5, -0.0] | 1.0 [-0.6, 2.7] | -0.5 [-2.5, 1.3] | | -1.8 [-3.8, 0.1] |  |
| Weighted | 1.1 [-0.0, 2.2] | -1.1 [-2.1, 0.2] | 1.4 [-0.2, 3.1] | -0.7 [-2.5, 1.2] | | -1.9 [-3.8, -0.0] |  |
|  | | | | | | | |
